# Bone marrow-derived mononuclear cells do not exert acute neuroprotection after stroke in spontaneously hypertensive rats

**DOI:** 10.3389/fncel.2013.00288

**Published:** 2014-01-08

**Authors:** Jens Minnerup, Daniel-Christoph Wagner, Jan-Kolja Strecker, Claudia Pösel, Sevgi Sevimli-Abdis, Antje Schmidt, Matthias Schilling, Johannes Boltze, Kai Diederich, Wolf-Rüdiger Schäbitz

**Affiliations:** ^1^Klinik für Neurologie, Department of Neurology, University of MünsterMünster, Germany; ^2^Fraunhofer Institute for Cell Therapy and Immunology and Translational Centre for Regenerative Medicine, University of LeipzigLeipzig, Germany; ^3^CATO Europe GmbHCologne, Germany; ^4^Massachusetts General Hospital and Harvard Medical SchoolCharlestown, MA, USA; ^5^EVK Bielefeld, Bethel, Neurologische KlinikBielefeld, Germany

**Keywords:** bone marrow-derived mononuclear cells, middle cerebral artery occlusion, stroke, dose-response, spontaneously hypertensive rats

## Abstract

Bone marrow-derived mononuclear cells (BM-MNCs) were shown to improve the outcome in animal stroke models and clinical pilot studies on BM-MNCs for stroke patients were already conducted. However, relevant aspects of pre-clinical evaluation, such as the use of animals with comorbidities and dose-response studies, were not thoroughly addressed so far. We therefore investigated different BM-MNC doses in the clinical meaningful stroke model of spontaneously hypertensive (SH) rats. Three hours after the onset of transient middle cerebral artery occlusion (MCAO) animals received either one of three syngeneic BM-MNC doses or placebo intravenously. The primary endpoint was the infarct size. Secondary endpoints included functional outcome, mortality, inflammatory processes, and the dose-response relationship. In contrast to previous studies which used healthy animals no beneficial effect of BM-MNCs was found. Infarct volumes, mortality, behavioral outcomes, and the extent of the inflammatory response to cerebral ischemia were comparable in all groups. In conclusion, we could not demonstrate that early BM-MNC treatment improves the outcome after stroke in SH rats. Whether BM-MNCs improve neurological recovery after delayed treatment initiation was not investigated in the present study, but our data indicates that this should be determined in co-morbid animal stroke models before moving to large-scale clinical studies. Future preclinical stroke studies on co-morbid animals should also include groups of healthy animals in order to determine whether negative results can be attributed to the comorbid condition.

## Introduction

Cell based therapies have been proposed as means of treating stroke. Among the different cell types under investigation for stroke, bone marrow-derived mononuclear cells (BM-MNCs) represent a particularly attractive treatment option. BM-MNCs can be easily obtained by bone marrow aspiration, do not require extensive preparation or cultivation, and permit autologous intravenous transplantation (Savitz et al., 2011b). Within the last several years, a number of reports showed that BM-MNCs reduce neurological impairment in animal models of focal cerebral ischemia after application in the acute and subacute phases (Iihoshi et al., 2004; Kamiya et al., 2008; Giraldi-Guimarães et al., 2009; Brenneman et al., 2010; Nakano-Doi et al., 2010). BM-MNC mediated neurological improvements including reduction of the final lesion volume, attenuated microglial activation, and promoted recovery after the initial ischemic event (Brenneman et al., 2010; Nakano-Doi et al., 2010; Sharma et al., 2010). Recent evidence suggests that BM-MNCs mediate their actions by the production and secretion of cytokines (Sharma et al., 2010). These in turn modulate the post-ischemic inflammatory response and attenuate neuronal cell death (Brenneman et al., 2010; Sharma et al., 2010; Wagner et al., 2012). Giving results from previous investigations reporting highest efficacy after BM-MNC transplantation within 3 h, early neuroprotective effects may be responsible for the majority of observed benefits (Iihoshi et al., 2004; Brenneman et al., 2010).

Recently, an open-label prospective study showed that intravenous BM-MNC treatment is safe and feasible in acute stroke patients (Savitz et al., 2011b). However, the pre-clinical package that is recommended to work up before proceeding to larger clinical efficacy studies has not been completed yet. For example, the *Stroke Therapy Academic Industry Roundtable* (STAIR) and *The Stem Cell Therapies as an Emerging Paradigm in Stroke* (STEPS) recommendations stress the importance of testing candidate cell therapies in animals with comorbidities (Fisher et al., 2009; Stem Cell Therapies as an Emerging Paradigm in Stroke Participants, 2009; Savitz et al., 2011a). Such studies are lacking for BM-MNC so far. This is remarkable given that the majority of stroke patients have comorbidities, such as hypertension. Moreover, analyzes of previous animal stroke studies revealed that experiments with healthy animals compared to those using animals with comorbidities overstated the efficacy of a given treatment (Crossley et al., 2008). Pre-clinical cell dose-response investigations are not only requested by the STAIR and STEPS guidelines but also recommended by the US Food and Drug Administration (FDA) to be performed prior to initiating a clinical trial.^1^

Here, we investigated the neuroprotective properties of systemically transplanted BM-MNCs on infarct size, functional outcome, and glial inflammatory processes as well as their dose-response relationship in spontaneously hypertensive (SH) rats with focal cerebral ischemia at 3 h following stroke, for which best efficacy was reported in a pervious study (Iihoshi et al., 2004).

## Experimental procedures

### Animals

All animal procedures were approved by the responsible ethics committee of the University of Münster and the appropriate authorities of the Federal State of North Rhine-Westphalia. The investigations were carried out in accordance with national and international animal welfare regulations and are reported in accordance with the *Animal Research: Reporting In Vivo Experiments* (ARRIVE) guidelines (Kilkenny et al., 2011). Surgery and evaluation of all read-outs were performed blinded to experimental groups. Experiments were performed on adult (12–13 weeks old) male SH rats weighing 260–290 g. SH rats were shown to have an increased blood pressure starting from 5 to 6 weeks of age (Dickhout and Lee, 1998). All animals were randomly assigned to one of the following treatment groups: (1) placebo (*n* = 19), (2) 1 million BM-MNCs/rat (*n* = 18), (3) 5 million BM-MNCs/rat (*n* = 20), or (4) 20 million BM-MNCs/rat (*n* = 17). The cell numbers used in our experiments were based on previous studies that investigated different intravenous cell therapies in animal stroke models (Iihoshi et al., 2004; Giraldi-Guimarães et al., 2009; Minnerup et al., 2011). The condition of animals was monitored at least every 8 h. Pre-defined termination criteria were: (1) a severe immobility and (2) a persisting abnormal body position. The implementation of these criteria was required by the local ethics committee.

### Bone marrow mononuclear cell preparation

Syngeneic rat bone marrow was obtained from male SH rats at the age of 12 weeks. Femurs and tibias were aseptically opened and repeatedly flushed with phosphate buffered saline (PBS). After erythrocyte lysis by ammonium chloride-based buffer (0.155 M NH_4_Cl, 10 mM KHCO_3_ and 0.01 mM Na_2_EDTA) cells were filtered by a 100 µm cell strainer, counted and prepared for immunomagnetic depletion of granulocytes: 100 µm million bone marrow cells were incubated with 10 ng/ml Phycoerythrin-conjugated anti-rat granulocyte antibody (clone RP1; BD Pharmingen, Heidelberg, Germany) for 15 min at 4°C. Subsequently, cells were washed with cold PBS plus 0.5% fetal calf serum (FCS) and incubated with 200 µl anti-Phycoerythrin MicroBeads (Miltenyi Biotech, Bergisch Gladbach, Germany) in 800 µl PBS plus 5% FCS for 15 min at 4°C. After incubation, non-adsorbed MicroBeads were removed by a further washing step. The cell suspension was then resuspended in 500 µl PBS plus 0.5% FCS and magnetically separated by a LD-column according to the manufacturer’s instructions (Miltenyi). This procedure results in higher BM-MNC purity compared to standard density gradient centrifugation (Pösel et al., 2012). The obtained mononuclear cell fraction was collected, counted, cryopreserved in liquid nitrogen (25 million mononuclear cells in 1 ml FCS plus 8% DMSO) and stored at −80°C until further use. BM-MNCs were labeled by PKH26 before transplantation (Zhang et al., 2011).

### Stroke model and cell therapy

Animals were anesthetized by intraperitoneal injection of ketamine hydrochloride (100 mg/kg body weight; Ketanest) and xylazine hydrochloride (8 mg/kg body weight). The rectal temperature was maintained at 37°C by a thermostatically controlled heating pad (Föhr Medical Instruments). Transient middle cerebral artery occlusion (tMCAO) was induced as described previously (Longa et al., 1989; Schäbitz et al., 2003). Briefly, following a midline neck incision, a 3–0 nylon filament (Ethicon) with a tip heated to form a bulb shape was inserted into the right common carotid artery and advanced via the internal carotid artery to occlude the origin of the right middle cerebral artery (MCA). Cerebral blood flow was monitored continuously to verify MCA occlusion and reperfusion using Laser-Doppler flowmetry (Periflux 5001; Perimed, Stockholm, Sweden) with a probe positioned over the MCA territory. After 60 min middle cerebral artery occlusion (MCAO), the filament was withdrawn to allow reperfusion. Three hours after onset of occlusion animals received the different cell doses or vehicle intravenously.

### Behavioral testing

After a 3 day training period, behavioral tests were performed during the light cycle 1 day before MCAO (baseline), and at 24 and 72 h after ischemia. Functional outcome was studied using the neurological score as described by Menzies (Menzies et al., 1992). This score ranges from 0, indicating no deficit, to 4, indicating a severe neurological deficit (spontaneous circling). For Rotarod tests, rats were placed on an accelerating Rotarod cylinder, and the time the animals remained on this cylinder was measured (Minnerup et al., 2010). Speed was increased from 4 to 40 rpm within 5 min. The trial ended if the animal fell off the rungs or gripped the device and spun around for two consecutive revolutions without attempting to walk on the rungs. An arbitrary time limit of 300 s was set for rats on the Rotarod cylinder in training and testing procedures. The mean duration (seconds) on the device was recorded with three measurements 1 day before surgery. For the adhesive-removal test, the somatosensory deficit was measured both before and after ischemia (Minnerup et al., 2010). Two small pieces of adhesive-backed paper dots of equal size were used as bilateral tactile stimuli occupying the palmar surface of each forepaw. The rat was then returned to a cage. The time to remove each stimulus from forelimbs was documented by three trials per day for each forepaw (Schneider et al., 2005). For the cylinder test, the rats were placed in a transparent cylinder (16 cm diameter, 21 cm height) and videotaped from underneath for 2 min (Minnerup et al., 2010). Spontaneous wall and ground touches of the impaired contralateral forelimb were counted.

### Determination of infarct size and immunohistochemistry

Three days after ischemia, animals were anesthetized and transcardially perfused with 4% paraformaldehyde in 0.1 mol/L phosphate buffer. The brains were fixed in 4% paraformaldehyde at 4°C and then cryoprotected in 30% sucrose solution. Tissue was stored at −80°C until analysis. For infarct size calculation, 10 µm serial coronal brain section were cut in a cryostat (Leica, Nussloch, Germany), collected at 800 µm intervals and stained with toluidine blue (Sigma, St. Louis, USA). Infarct volumes were quantified with a standard computer assisted image analysis technique. To correct for the effect of brain edema, a correction method was used as described previously (Sevimli et al., 2009). For histological evaluation of inflammation, immunohistochemistry was performed on glass mounted coronal brain sections (10 µm, approx. bregma −1 mm). After blocking of non-specific proteins (using blocking reagent, 15 min, Roche Diagnostics, Mannheim, Germany) the following primary antibodies were applied (overnight, 4°C): anti-GFAP (raised in mouse, 1:500, Dako, Hamburg, Germany) and anti-Iba1 (raised in goat, 1:200, Abcam, Cambridge, UK). To detect anti-GFAP-antibodies we used a biotinylated goat anti-mouse antibody (1:100, Jackson Labs, West Grove, PA, USA) for 45 min at room temperature. Astrocytes were visualized by a streptavidin conjugated fluorescent dye (AlexaFluor594, Molecular Probes, Leiden, the Netherlands). For signal amplification of the Iba1 signal, brain slices were pre-treated with 3% H_2_O_2_/Methanol for 10 min in order to block endogenous peroxidase. Detection of Iba1-antibodies was performed using a biotin conjugated donkey anti-goat antibody (1:200, 45 min, room temperature, Jackson Labs, West Grove, PA, USA), followed by incubation with horseradish peroxidase/streptavidin (1:100, 45 min, DAKO, Glostrup, Denmark) and biotinyl tyramide, (1:100) for 10 min at room temperature. Iba1-positive cells were visualized using AlexaFluor594. A fluorescent-preserving mounting medium containing 4′, 6-diamidino-2-phenylindole (DAPI) was used for nuclear counterstaining (Vector, Burlingame, CA, USA). The immunofluorescence signal was visualized with a fluorescent microscope (Nikon Eclipse 80i microscope, Nikon GmbH, Duesseldorf, Germany) with appropriate filter sets for AlexaFluor594 and DAPI. Digitizing was done with a Stereo Investigator Software (MicroBrightField Inc., Williston, VT, USA). To determine whether administered bone marrow-derived mononuclear cells have an influence on the post-ischemic reactive astrogliosis, GFAP-stained sections (three randomly chosen animals per group) were analyzed with respect to differences in morphology and immunoreactivity between the investigated groups. Quantification of Iba1-posive cells was performed by counting and averaging absolute cell amounts covering four random fields (0.25 mm^2^ each) within the ipsilateral ischemic core and the boundary zone of the infarct of two separate brain sections per animal.

### Primary and secondary objectives of the study

According to the ARRIVE guidelines primary and secondary objectives were defined.^2^ The primary endpoint was infarct size measured on day 3 after ischemia. Secondary endpoints were the functional outcome, mortality between treatment and day 3 (when animals were killed), inflammatory processes, and the dose-response relationship.

### Statistical analysis

Values are presented as mean ± SD. The sample size (number of animals per group) was calculated *a priori* with the following assumptions: (1) infarct size reductions of ≥20% should be detected, (2) a power of 0.8, (3) an α of 0.05, and (4) a SD 15% of the mean. A one-way analysis of variance (ANOVA) with Tukey’s post-hoc test was used to compare of infarct volumes, inflammatory processes, and physiological measures. Mortality was compared using the chi-square test. Behavioral tests were analyzed with two-way repeated-measures ANOVA. Statistical significance was determined as an α error <0.05. Statistical analyses were carried out using the Statistical Package of Social Sciences (version 18).

## Results

### Mortality and physiological measurements

There was no statistically significant difference in mortality between the four groups. A total of 11 animals died until day 3: Three of the placebo group, 2 of the 1 million BM-MNCs/rat group, 4 of the 5 million BM-MNCs/rat group, and 2 of the 20 million BM-MNCs/rat group (*P* = 0.859). None of the animals met any of the termination criteria. The animals died spontaneously due to the cerebral ischemia. Rectal temperature, body weight, and cerebral blood flow measurements were not different between groups (all *P*-values > 0.05).

### Infarct volumes

**Figure 1:**
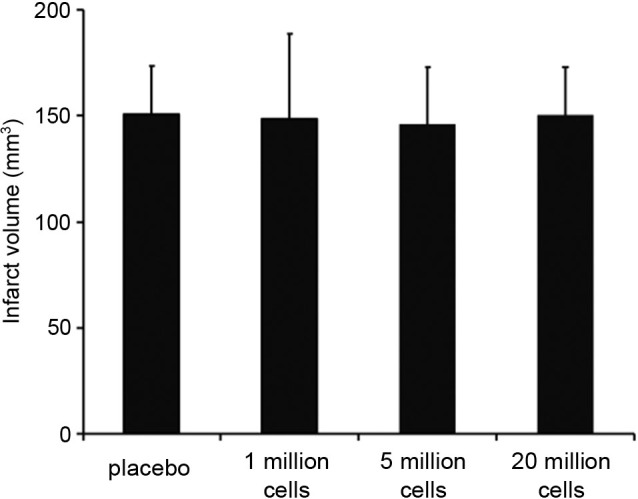
**Infarct volumes 3 days after transient MCAO**. Infarct volumes did not differ between the four groups (*n* = 15–16/group). *P* = 0.595.

Infarct volumes did not differ between the four groups (placebo: 150.55 mm^3^ ± 23.11 mm^3^, 1 million BM-MNCs/rat: 148.74 mm^3^ ± 39.90 mm^3^, 5 million BM-MNCs/rat: 145.59 ± 27.52 mm^3^, 20 million BM-MNCs/rat: 150.55 mm^3^ ± 23.11 mm^3^; *P* = 0.595; Figure 1).

### Functional outcome

**Figure 2:**
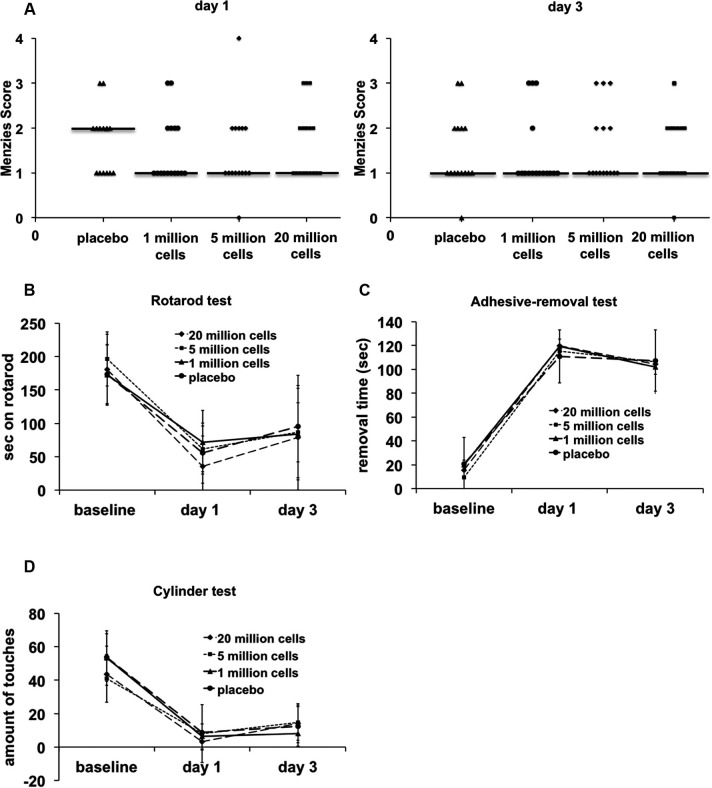
**Behavioral testing until day 3 after transient MCAO**. No significant differences between the groups were detected in any of the functional tests (*n* = 15–16/group). **(A)** Menzies score, *P* = 0.980. **(B)** Rotarod test, *P* = 0.670. **(C)** Adhesive-removal test, *P* = 0.922. **(D)** Cylinder test, *P* = 0.350.

Functional outcomes as assessed by a behavioral test battery were not improved in any of the BM-MNC treated groups (Figure 2) (Menzies score: *P* = 0.980, Rotarod test: *P* = 0.670, adhesive-removal test: *P* = 0.922, cylinder test: *P* = 0.350).

### Analysis of transplanted Bone marrow-derived mononuclear cells (BM-MNCs) and post-schemic inflammation

**Figure 3:**
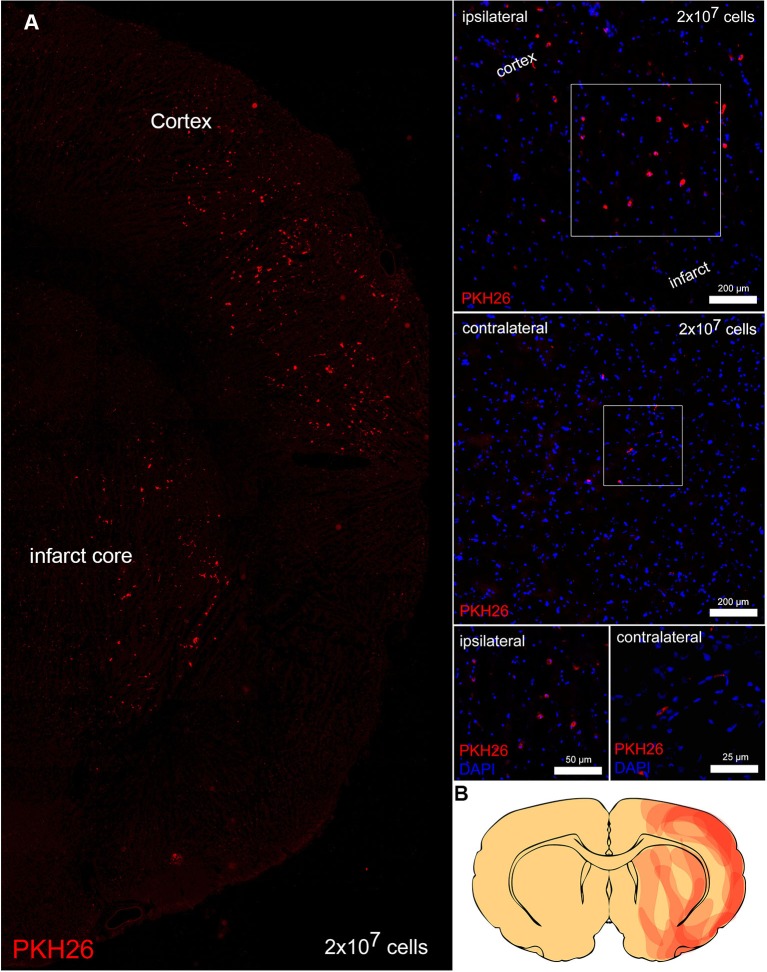
**Transplanted cells in the brain. (A)** Transplanted PKH26 positive (red) BM-MNCs in the boundary zone of the infarct and in the infarct core. Ipsilateral to the infarct BM-MNCs were located in the parenchyma, whereas contralateral PKH26 positive cells were only detected in vessels. Overall, only a few PKH26 positive cells were detected contralateral. Nuclei were counterstained with 4′, 6-diamidino-2-phenylindole (DAPI, blue). **(B)** The map depicts immigrated PKH26 positive cells within the infarcted hemisphere (obtained from five animals).

Transplanted, PKH26 labeled cells were predominantly located on the ischemic side of the brain and were sparsely detected at the contralateral side. On the ischemic side, PKH26 positive cells were located within the boundary zone of the ischemic lesion (Figure 3). On the contralateral side BM-MNCs were only detected in vessels.

**Figure 4:**
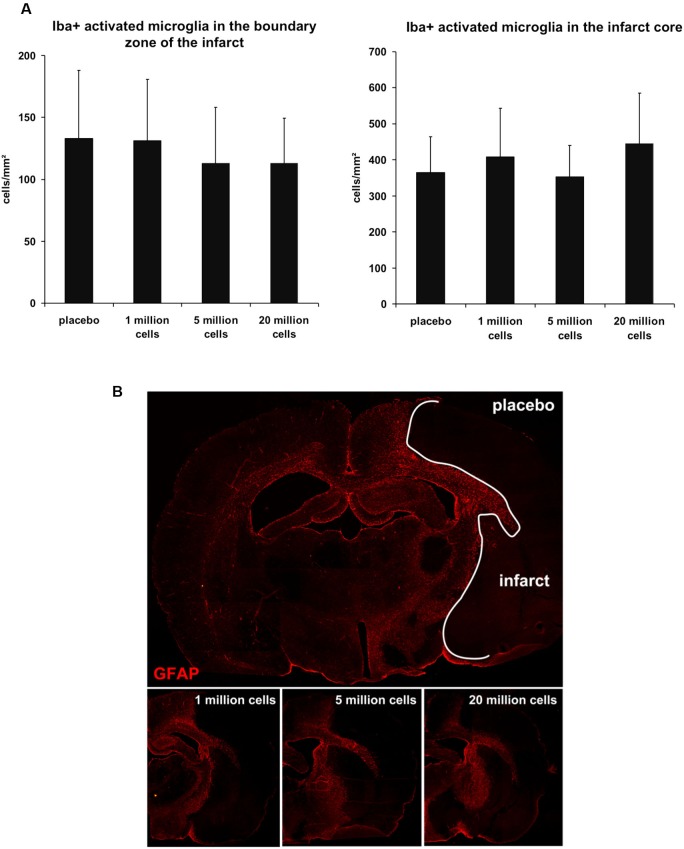
**Cellular inflammatory response 3 days after MCAO. (A)** There was no statistical difference between the groups regarding the number of activated microglia cells (Iba1-positive) within the infarct core (*P* = 0.122) and in the boundary zone (*P* = 0.476) of the infarct (*n* = 15–16/group). **(B)** GFAP staining visualized the common distribution of astrogliosis surrounding the infarcted tissue. No differences with respect to GFAP-immunoreactivity and morphology between the BM-MNC and the placebo treated groups were observed (*n* = 3/group).

Inflammation contributes to ischemic brain damage and BM-MNCs were assumed to exert neuroprotective effects via anti-inflammatory mechanisms (Dirnagl et al., 1999). We therefore analyzed the extent of the glial inflammatory response. The number of Iba1-positive cells with retracted processes and round cell bodies, representing activated microglia, did not significantly differ within the infarct core (*P* = 0.122) and within the boundary zone of the infarct (*P* = 0.476) between the different groups (Figure 4A). Pattern of astrocyte activation visualized by GFAP-immunoreactivity was also comparable among all groups (Figure 4B).

## Discussion

The present study did not show a beneficial effect for either dose of BM-MNCs given intravenously 3 h after the onset of focal cerebral ischemia in SH rats. There were no significant effects on the primary and secondary end points. Infarct volumes, mortality, behavioral outcomes, and the extent of the inflammatory response to cerebral ischemia were comparable in the placebo and in all BM-MNC groups. Particularly, no dose-response relationship of BM-MNC treatment was found. Our results indicate that BM-MNCs do not exert acute neuroprotective properties in SH rats although transplanted BM-MNCs reached the brain.

The efficacy of early BM-MNC treatment initiation within 6 h after stroke onset was investigated by a number of studies (Iihoshi et al., 2004; Baker et al., 2007; Kamiya et al., 2008). For intravenous cell administration, a higher efficacy of early BM-MNC therapy at 3 h following stroke was shown as compared to later treatment (Iihoshi et al., 2004). Moreover, recombinant tissue-type plasminogen activator (rt-PA) treatment was approved until 3 h after the onset of ischemia at the time when the present study was designed (Hacke et al., 2008). Hence, the time for cell therapy initiation seems to be reasonable from a translational perspective. However, the sustained therapeutic benefit of early intravenous BM-MNC application could not be reproduced in our investigation, with the use of healthy animals in aforementioned experiments being the major difference in study design. Indeed, contrary results on a treatment’s efficacy depending on the animal strain used in a study with greater infarct size reductions in healthy animals compared to animals with comorbidities were previously reported (Crossley et al., 2008). This observation was even assumed to contribute substantially to the translational failure in experimental stroke research (Crossley et al., 2008). However, despite the use of hypertensive animals in our study and the use of healthy animals in previously published positive studies is the most obvious difference, our study does not provide the final proof that the hypertensive condition of the host or of the donor is the reason for the negative results, since no healthy, non-hypertensive control group was included. Other reasons such as the preparation of the BM-MNCs or the way of cell transplantation might also be relevant for our negative results.

Baker et al. (2007) who also used healthy animals, found reduced infarct volumes and improved functional outcomes after early BM-MNC treatment (Baker et al., 2007). The intraarterial administration of BM-MNCs in this study is a further main difference to our experiments which might explain the contrary results. Although intraarterial procedures after stroke are getting more common and safer, results from a pre-clinical study found that intraarterial cell therapy is associated with an increased mortality (Walczak et al., 2008; Saver et al., 2012). From a translational perspective, intravenous approaches therefore seem to be the first choice in animal models and early-stage clinical trials (Savitz et al., 2011b). So far, one previous study suggested that systemic BM-MNC treatment may not be beneficial when initiated after the onset of ischemia (Kamiya et al., 2008). The significance of this study is, however, limited as the number of animals was rather small (*n* = 5) and only one cell dose was evaluated.

Therapies with cells of different sources were demonstrated to exert anti-inflammatory actions after cerebral ischemia (Lee et al., 2008; Schwarting et al., 2008). With respect to BM-MNC treatment, immunomodulatory mechanisms post-stroke were suggested based on *in vitro* investigations and on studies which found a BM-MNC treatment associated reduction of proinflammatory cytokines (Brenneman et al., 2010; Sharma et al., 2010). Our experiments, which investigated the local microglial immune response, however, did not confirm that BM-MNCs reduce this important element of post-stroke inflammation. A possible explanation for these apparently different observations might be that in the mentioned study cytokine levels of the whole brain were measured (Brenneman et al., 2010). Since healthy animals were used, infarct sizes were smaller after BM-MNC treatment and this reduced volume of injured tissue in turn might be the true reason for the lower cytokine concentrations.

Our study has strengths and limitations. The experiments rigorously adhered to stringent quality criteria in experimental stroke research such as randomization, surgery and evaluations performed in a blinded fashion, and controlled physiological parameters (Dirnagl, 2006; Kilkenny et al., 2011). Additionally, the use of animals with a relevant comorbidity may increase the predictive value of our findings regarding a human stroke patient population. Studies with negative results have a potential general weakness, i.e., the type II error. The type II error means that the null hypothesis is not rejected despite being false. Applied to the present study BM-MNCs in fact would reduce infarct volumes while not doing so in our experiments. However, we performed a thorough *a priori* sample size calculation to detect infarct size reductions of ≥20% with a statistical power of 0.8 which corresponds to a type II error of 0.2. Moreover, the use of a range of BM-MNC numbers should have prevented to miss the efficacy of a specific cell dose. Another potential limitation of our study is the generalizability. We cannot rule out that an alternative procedure for cell preparation or the use of healthy animals, animals of another strain or sex might have yield different results. Our negative finding is therefore restricted to the cell preparation, the route of transplantation and the animal model used in this study. Particularly, the negative results cannot attributed to the co-morbid condition of the animals with certainty, since no control groups of healthy animals were included.

Although neutral and negative experimental results are disappointing at a first glance publishing those is certainly of great importance when considering the tremendous costs of clinical studies and the potential risks for patients. The present results add relevant information to the pre-clinical development process of BM-MNCs for stroke therapy. While using a clinically meaningful model for post-stroke neuroprotection, we showed that BM-MNCs do not improve outcome when given in the early phase after the onset of ischemia. This may affect upcoming efficacy studies in patients based on the rational that early induction of BM-MNC therapy may lead to more favorable outcomes.

Indubitably, previous animal experimental stroke studies found improvements after BM-MNC therapy (Giraldi-Guimarães et al., 2009; Brenneman et al., 2010; Nakano-Doi et al., 2010). However, these studies initiated treatment beyond established time windows of acute neuroprotection, thus potentially aiming to restore neurological function by promoting regeneration rather than protecting tissue at risk. Indeed, BM-MNCs can also promote neurological recovery by enhancing neurogenesis and angiogenesis in later phases and were demonstrated to stimulate brain remodeling processes, such as the proliferation of neural progenitor and endothelial cells, and to reduce neurodegeneration (Giraldi-Guimarães et al., 2009; Nakano-Doi et al., 2010). Since the investigation of these later effects was not in the focus of our investigation, a direct conclusion on the impact of BM-MNC treatment at later stages following experimental stroke may not be valid, but remains for further study. However, the remarkable differences regarding acute neuroprotection in healthy and comorbid animals as revealed by the present study clearly underline the necessity to evaluate relevant treatment effects and the optimal treatment time window in animal models more adequately reflecting the situation of stroke patients. This will help to prepare large scale efficacy studies with maximum rigor regarding our knowledge on therapeutic mechanisms, and thereby efficacy in human patients.

## Conflict of interest statement

The authors declare that the research was conducted in the absence of any commercial or financial relationships that could be construed as a potential conflict of interest.

## Footnotes

^1^http://www.fda.gov/cber/guidelines.htm, 2012.

^2^http://www.nc3rs.org/ARRIVE, 2012.
